# Detection of single-nucleotide polymorphisms in growth hormone and insulin-like growth factor-1 genes related to growth traits in purebred and crossbred quails

**DOI:** 10.14202/vetworld.2024.1482-1489

**Published:** 2024-07-10

**Authors:** Mohamed H. Khalil, Eman A. Elattar, Ayman S. EL-Seedy, Mostafa K. Shebl

**Affiliations:** 1 Department of Poultry Production, Faculty of Agriculture (El-Shatby), Alexandria University, Alexandria, Egypt; 2 Laboratory of Cellular and Molecular Genetics, Department of Genetics, Faculty of Agriculture (El-Shatby), Alexandria University, Alexandria, Egypt

**Keywords:** crossing effects, genes for growth hormone and insulin, growth, polymorphism, quail

## Abstract

**Background and Aim:**

There is a limited amount of research conducted on quail breeding domestically and internationally, particularly at the molecular level. This study aimed to detect single-nucleotide polymorphisms in the growth hormone (GH) and insulin-like growth factor-1 (IGF-1) genes across two quail varieties and their hybrids correlate these genetic factors with body weight (BW) and growth rate at 0 and 6 weeks, and assess crossing effects.

**Materials and Methods:**

White and Japanese quail were crossed. Simultaneously producing pure varieties and crosses (genotypes) was achieved through this breeding strategy. Fifty females from each genotype were randomly selected for blood sampling. Genomic DNA was extracted and amplified from the blood using the DNeasy blood kit (Qiagen, Germany). Nucleotide polymorphism between quail genotypes was determined through DNA sequencing.

**Results:**

Two types of alleles (A and B) for the GH gene in quails showed significant genotypic differences (AA, BB, and AB). The quail carried a mutated IGF-1 gene. For growth traits, substantial positive heterosis was detected.

**Conclusion:**

The genotype AA had the highest BW and weight gain. The white variety can act as a sire, and both white and Japanese varieties can function as dams to improve growth traits. The growth characteristics of the hybrids surpassed those of the original varieties.

## Introduction

The smallest bird species raised for meat and eggs is the Japanese quail. The Japanese quail is the finest model species in breeding experiments because of its short generation intervals [1]. Although quail output is much smaller than that of broiler chickens in the poultry world, quail is still a valuable meat source, plays an important role in poultry breeding, and supports the global poultry industry [2].

The application of molecular genetics techniques has significantly boosted farm animal breeding improvement programs’ effectiveness. In quails, genes responsible for economic traits like meat production could vary. The screening of single-nucleotide polymorphisms (SNPs) inside candidate genes and their application as marker-assisted selection was applied for productive traits evaluation and greatly impacted the productivity of studied birds of quail [3–10]. Accordingly, two candidate genes were reported as growth hormone (GH) and insulin-like growth factor-1 (IGF-1) genes [6]. Therefore, the associations of SNPs with meat production performance in quail birds will help to detect the role of genetic factors in meat traits. Crossbreeding programs are commonly used in commercial hen breeding systems to utilize heterosis [11]. Developing a novel hybrid line is critical for obtaining high-growth broilers and high-laying hen productivity.

This study aimed to identify gene polymorphisms in the GH and IGF-1 genes, determine relationships between the identified genotypes and growth traits, and estimate crossing effects for growth traits in a quail (White and Japanese) crossing experiment.

## Materials and Methods

### Ethical approval

Since non-intrusive methods were used, obtaining Institutional Animal Ethics Committee consent for this investigation was unnecessary. However, birds were handled without any harm and unnecessary stress.

### Study period and location

During the breeding season of October 2018 to July 2019, the experimental work for this study was conducted at the Poultry Research Center, Poultry Production Department, Faculty of Agriculture, Alexandria University, Egypt. Molecular Genetics was performed at the Faculty of Agriculture, Department of Cellular and Molecular Genetics, Alexandria University. Two varieties (genotypes) of quails, White (WW) and Japanese (JJ), were crossed.

### Scheme for mating

A total of 160 families (40 families per genotype) were randomly distributed in 160 cages with a sex ratio of 1:2. The breeding strategy allowed for the concurrent production of pure varieties (W × W, J × J) and crosses (W × J, J × W). First on the list of crosses was the male parent. Except for avoiding sib-mating, mating within the varieties (genotypes) was random. Six weekly hatches produced offspring of reciprocal crosses and pure varieties.

### Flock husbandry

The chicks were pedigreed for each family at hatching, found in floor brooders, wing banded, and brooded. Once the females reached 6 weeks of age, they were assigned to individual laying cages. Water and food were always available. The crude protein content of the diet was 24% until 6 weeks of age and 20% after 6 weeks. From the time, the pullets were 6 weeks old until the end of the experiment, they were exposed to light for 16 h every day. All birds received the same care and medication throughout the experimental period under the same administrative and environmental conditions.

### Studied traits

For both sexes, the following growth traits were individually recorded:

- Body weight (BW) in grams (g) at hatching and 6 weeks of age (BW)
- Body weight gain (BWG) in grams (g) between weeks 0 and 6 of age (BWG).

### Molecular genetic analysis

#### Blood sampling and DNA extraction

Fifty females from each variety were assigned randomly to collect quail blood samples. One milliliter of blood was extracted from each wing vein. Using the DNeasy Blood Kit (Qiagen, Germany), genomic DNA was extracted from blood according to the manufacturer’s instructions. DNA was maintained at −20°C until use. The quality of the extracted DNA was assessed by agarose gel electrophoresis on a 1.5% agarose gel with ethidium bromide in a 1× Trisacetate-EDTA (TAE) buffer and an ultraviolet spectrophotometer (Jenway, UK) was used to evaluate the amount, followed by separation using primers synthesized by commercial service providers.

#### Polymerase chain reaction (PCR) conditions

The PCR reaction was conducted in a 50-μL volume using 10× PCR buffer (50 mMl/l KCl, 10 mM/l Tris–HCl [pH 8.0], 0.1% Triton X-100) 5 μL, 1.5 mM MgCl_2_ 3 μL, 0.2 mM each dNTP (deoxynucleotide triphosphate) 8 μL, 10 pM/L each primer 1 μL, 100–150 ng genomic DNA, and 5U Taq DNA polymerase. The following were the PCR conditions: Denaturation at 94°C for 5 min followed by 35 cycles of denaturation at 94°C for 1 min, annealing at 62°C for 1 min, extension at 72°C for 1 min, and final extension 72°C for 10 min, on Watercycle® Gradient (Eppendorf AG, Hamburg, Germany).

#### Genotyping

All PCR products were separately digested using MspI, AccI, and BsrDI restriction enzymes for the selected genes. The PCR product was present in 20 μL of the 30 μL volume used for the digesting step, bovine serum albumin (100 μg/mL) 0.3 μL, Buffer 1 × 3 μL, 2 h at 37°C, and then, a 20-min deactivation procedure led at 65°C. The resulting fragments were separated by electrophoresis on a 2% agarose gel in parallel with a 100-bp DNA marker. Genotyping will be performed on all samples.

#### Sequence analysis

After purification in both directions using Dye Terminator 3.1 chemistry (Thermo Fisher Scientific, UK), DNA fragments from specific samples were sequenced to identify the sites of nucleotide substitutions (SNPs) responsible for gene polymorphisms. These sequences were compared for the presence of SNPs.

### Growth gene (GH)

#### PCR amplification

The primer pairs used to amplify GH gene are listed in Table-1. PCR was performed with the program beginning pre-denaturation conditions for 5 min at a temperature of 95°C, denaturation for 30 s at a temperature of 94°C, annealing at 56°C for 45 min, elongation at 72°C for 1 min, 35 cycles, and final extension at 72°C for 5 min.

**Table-1:** Primer sequence of genes and restriction enzymes.

Gene	Primer sequence of the genes (forward and reverse)	Restriction enzymes
GH-1	GH-F 5’-ATCCCCAGGCAAACATCCTC-3’ GH-R 5′-CCTCGACATCCAGCTCACAT-3′	MspI
IGF-1	IGF-F5’- TTTGCCAGAAGAGGGAGAGA-3’ IGF-R5’- GCAGAAGCAGACAACACACA-3’	AccI

GH=Growth hormone, IGF=Insulin-like growth factor

The PCR products were digested with the enzyme MspI. Polymorphism detection was conducted in two stages: (a) identification of genotypes of the GH gene and (b) calculation of allele frequencies to determine the relationship between divergent selection BWs and production performance with the resulting genotypes.

#### Finding polymorphisms in GH

The digestion profile revealed polymorphisms in the GH gene fragment (776 bp in size). The goal of quail research was to generate two different types of alleles (A and B). Every sample’s electrophoresis was compared with the DNA marker bands. Polymorphisms in the GH gene were indicated by a single band: 776 bp (genotype BB), two bands: 539 bp and 237 bp (genotype AA), or three bands: 776 bp, 539 bp, and 237 bp (genotype AB).

### Gene for IGF-1

#### PCR amplification

The primer pairs used to amplify IGF-1 gene are listed in Table-1. PCR was performed using the T-professional thermal cycler (Biometra, Germany) and DreamTaq Green PCR Master Mix (Thermo Scientific). Each reaction mixture comprised 25 μL of the master mix, 2 μL of the DNA template, 1.5 μL of each primer (10 pmol/μL), and some deionized water to have a final volume of 50 μL. Cycles applied were pre-denaturation conditions for 3 min at 94°C, denaturation for 20 s at 94°C, annealing at 53°C for 45 min, elongation at 72°C for 30 s, 37 cycles, and final extension at 72°C for 7 min. The PCR products were screened by 1.5% agarose gel electrophoresis in 1 × TAE buffer and were visualized in a gel documentation system with a transilluminator (Syngene, UK).

### Statistical analysis

Data were examined for genotype-to-genotype variance using IBM Statistical Package for the Social Sciences 28.0 software (IBM Corp., NY, USA) using a general linear model [12]. Duncan’s test was used to determine the significance of the differences [13]. To analyze the data of growth traits at 6 weeks of age, the following linear model was tested:

Y_ijkl_ = U + G_i_ + H_j_ + S_k_ + GH_ij_ + GS_ik_ + HS_jk_ + GHS_ijk_ + e_ijkl_

Where: Y_ijkl_ = The observation of the genotype, U = The overall mean, G_i_ = The fixed effect of i^th^ genotype, H_j_ = The fixed effect of the j^th^ hatch, S_k_ = The fixed effect of k^th^ sex, GH_ij_, GS_ik_, HS_jk_, GHS_ijk_ = Fixed effects interaction, and e_ijkl_ = Residual error.

Data of BW at hatch were examined using the prior general linear model for BW at 6 weeks of age, excluding sex impact from the model.

#### Estimation of crossbreeding components

Estimates of direct additive effect, direct heterosis, and reciprocal effect for growth traits were calculated according to Dickerson [14, 15] using IBM SPSS Statistics 28 Software Package [12].

1. Pure strain difference:
  [(WW × WW–JJ × JJ)]
2. Direct additive effect:
  [(WW × WW + WW × JJ) – (JJ × JJ + JJ × WW)]/2
3. Direct heterosis:
  [(WW × JJ + JJ × WW) – (WW × WW + JJ × JJ)]/2
4. Reciprocal effect:
  [(JJ × WW) – (WW × JJ)]/2

The following formula was used to compute heterosis (H%) [16].

H% = [F_1_−(P_1_+P_2_)/2]/[(P_1_+P_2_)/2] × 100

Where: F_1_: average values of traits of hybrid varieties (genotypes).

P_1_, P_2_: average values of traits of the original varieties (genotypes).

## Results

### Detection of candidate gene polymorphisms (GH, IGF-1) in the genotypes and estimation of the association between the identified genes and growth characteristics

#### GH gene

**Figure-1:**
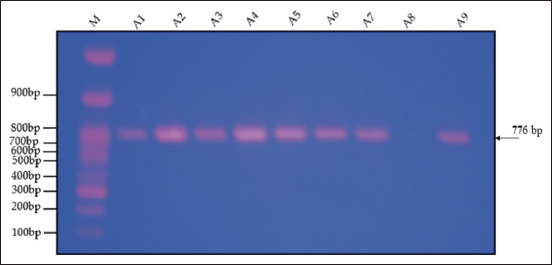
Polymerase chain reaction amplification of the growth hormone gene in the selected line of Japanese quail. M: 100-bp ladder.

PCR shows the amplification of the GH gene in Japanese quail, M: 100-bp ladder. The product of the GH gene was 776 bp, as distinguished by 1.5% agarose gel electrophoresis (Figure-1).

### Detection of GH gene polymorphism

**Figure-2:**
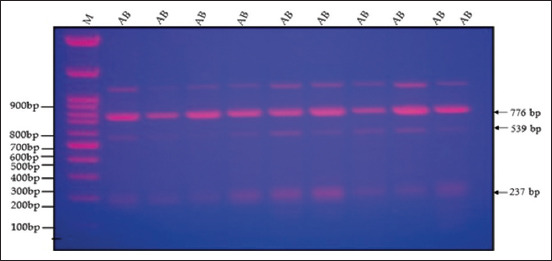
Polymerase chain reaction-restriction fragment length polymorphism band pattern of the growth hormone gene on a 3% agarose gel for genotype (AB).

**Figure-3:**
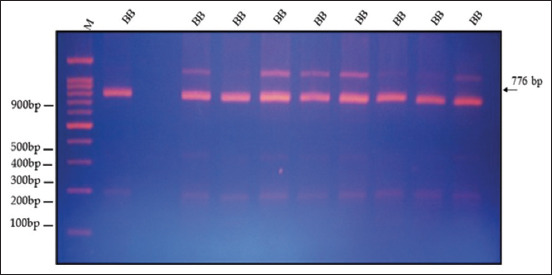
Digested PCR product of the growth hormone gene using MspI in PCR-RFLP analysis after electrophoresis on 3% agarose gel for the genotype (BB). PCR-RFLP=Polymerase chain reaction-restriction fragment length polymorphism.

**Figure-4:**
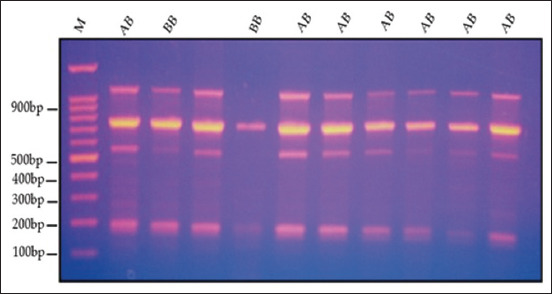
Digested PCR product of the growth hormone gene using MspI in PCR-RFLP analysis after electrophoresis on 3% agarose gel for the genotypes (AB) and (BB). PCR-RFLP=Polymerase chain reaction-restriction fragment length polymorphism.

**Figure-5:**
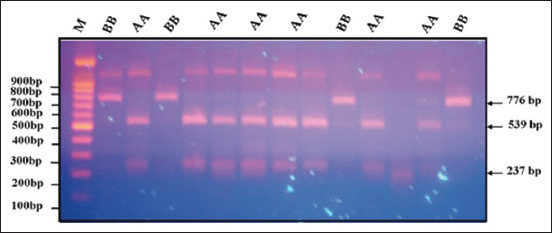
Polymerase chain reaction-restriction fragment length polymorphism band pattern of the growth hormone gene on a 3% agarose gel for the genotypes (AA) and (BB).

Figures 2–5 display the PCR product utilizing MspI in PCR-restriction fragment length polymorphism (PCR-RFLP) analysis following electrophoresis on 3% agarose gel. The results of the association analysis between different genotypes and growth traits of two varieties of quails, White (WW) and Japanese (JJ), are shown in Figures-2–5. For cross (W × J) and reciprocal cross (J × W) quails, there were two types of alleles (A and B) with significant differences between genotypes (AA, BB, and AB) for the growth traits studied (Table-2).

**Table-2:** Body weight at six weeks and body weight gain (0–6) weeks (Means ± standard error) by genotype of Growth hormone gene (GH gene) and frequency by cross in quail.

Genotypes for GH gene	Band No.	Cross (W x J)			Reciprocal Cross (J x W)			(W x J) vs. (J x W) Sig.	
		
		N (genotype frequency)	BW at 6 weeks (g)	BWG at (0-6) weeks (g)	N (genotype frequency)	BW at 6 weeks (g)	BWG at (0-6) weeks (g)	BW	BWG
AA	237 bp	30 (0.60)	333.55^a^ ± 2.98	324.17^a^ ± 2.99	16 (0.32)	306.61^a^ ± 2.10	297^a^ ± 2.10	****	****
	539 bp								
AB	237 bp	15 (0.30)	275.44^b^ ± 1.07	266.77^b^ ± 1.07	27 (0.54)	263.97^b^ ± 1.22	254.29^b^ ± 1.21	****	****
	539 bp								
BB	776 bp	5 (0.10)	232.40^c^ ± 0.96	223.76^c^ ± 0.95	7 (0.14)	227.12^c^ ± 1.2	217.99^c^ ± 1.19	****	****
	776 bp								
Sig.			****	****		****	****		

**** p ≤ 0.0001, BW=Body weight, BWG=Body weight gain, GH=Growth hormone, W × J = White male × Japanese female J × W= Japanese male × White female, (a, b, c): Different letters within a column indicate statistically significant differences

Figure-2 shows different genotypes for growth traits with the existence of polymorphisms in the GH gene fragment at 776, 539, and 237 bp. Electrophoresis of each sample was compared with the DNA marker bands and showed three bands of 237, 539, and 776 bp for the genotype (AB). (Figure-3) showed a single band of 776 bp for the genotype (BB). (Figure-4) displayed a single band of 776 bp for the genotype (BB) and three bands of 237, 539, and 776 bp for the genotype (AB). (Figure-5) displayed a single band of 776 bp for the genotype (BB) and two bands of 237 and 539 bp for the genotype (AA).

Table-2 presents the means of BW at 6 weeks and BWG at (0–6) weeks for quail based on genotype groups for the GH gene by the cross. For the cross (W × J), the genotypic frequencies of the GH genes (AA, AB, and BB) were 0.6, 0.3, and 0.1, respectively. The corresponding frequencies of the GH gene genotypes for the cross (J × W) were 0.32, 0.54, and 0.14, respectively.

Regarding the cross (W × J), a comparison between the genotypes of the GH gene (AA, AB, and BB) determined that the genotype (AA) in BW at 6 weeks and BWG at 0–6 weeks of age was substantially the best with mean of 333.55 and 324.17 g, respectively, followed by the GH genotype (AB) with mean of 275.44 and 266.77 g. The GH genotype (BB) was statistically the least BW and BWG (232.40 and 223.76 g, respectively). The same pattern was noted for the cross (J × W). In comparison between both crosses (W × J and J × W), the cross (W × J) was significantly better in BW and BWG than the reciprocal cross (J × W) for all genotypes of the GH gene (AA, AB, and BB).

### IGF-1 gene

**Figure-6:**
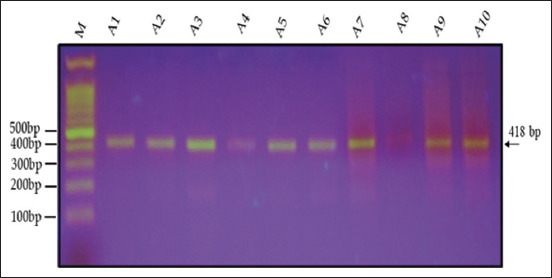
Electrophoresis of the 418-bp fragment of insulin-like growth factor-1 in white quail on 1.5% agarose gel. M: 100-bp ladder.

**Figure-7:**
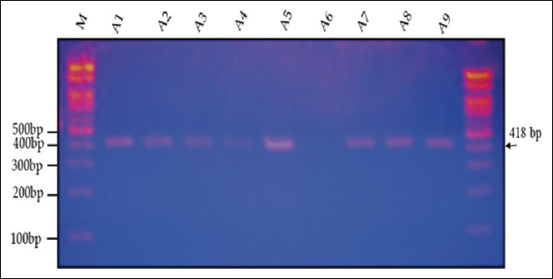
Electrophoresis of the 418-bp fragment of insulin-like growth factor-1 in Japanese quail on 1.5% agarose gel. M: 100-bp ladder.

**Figure-8:**
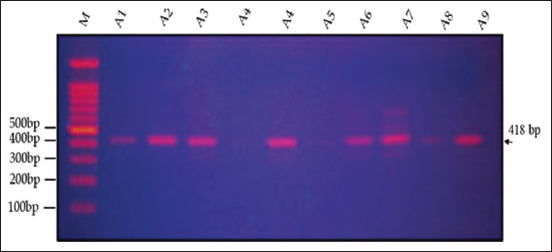
Electrophoresis of the 418-bp fragment of insulin-like growth factor-1 in cross of quail (W × J) on 1.5% agarose gel. M: 100-bp ladder.

**Figure-9:**
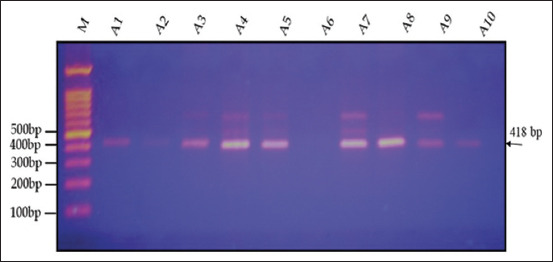
Electrophoresis of the 418-bp fragment of insulin-like growth factor-1 in reciprocal cross (J × W) of quail on 1.5% agarose gel. M: 100-bp ladder.

Agarose gel electrophoresis (1.5%) revealed that the amplification product of the IGF-1 gene, as determined by PCR, was 418 bp in two varieties (genotypes) of quails, White (WW) and Japanese (JJ), and their cross (W × J) and reciprocal cross (J × W) (Figures-6–9). A single band of 418 bp of the IGF-1 gene was observed for the purebred (WW and JJ) and crossbred (W × J and J × W) genotypes.

### Crossing effects of quails on growth traits

#### BW and BWG

Purebreds difference

Significant differences in BW were observed between genotypes at all ages studied (Table-3). The purebred genotype (WW) was significantly heavier at 6 weeks of age and grew faster during the period (0–6 weeks) than the other purebred (JJ) or crossbred genotypes (WJ and JW). In addition, the crossbreds (WJ and JW) were significantly heavier and grew faster than the purebred (JJ). BW means at 6 weeks of age were 270.27, 245.03, 260.18, and 257.56 g for the genotypes (WW, BB, WB, and BW), respectively. The corresponding BWG values during the growth period (0–6 weeks) were 261.13, 236.55, 251.49, and 248.09 g. At 6 weeks of age, BW and BWG were not significantly affected by gender.

**Table-3:** Means and SE of growth traits by mating type and sex and crossing analysis.

Mating type	Growth traits					

	Body weight at hatch (g) (BW0)		Body weight at 6 weeks (g) (BW6)		Body weight gain (0–6) weeks (g) (BWG)	
		
	n	Means ± SE	n	Means ± SE	n	Means ± SE
W × W (White)	326	9.10^b^ ± 0.06	214	270.27^a^ ± 2.13	214	261.13^a^ ± 2.12
J × J (Japanese)	649	8.39^d^ ± 0.04	398	245.03^c^ ± 1.55	398	236.55^c^ ± 1.55
W × J	734	8.55^c^ ± 0.04	439	260.18^b^ ± 1.42	439	251.49^b^ ± 1.42
J × W	419	9.38^a^ ± 0.05	265	257.56^b^ ± 1.87	265	248.09^b^ ± 1.86
Sig.		****		****		****
Sex						
Male			611	253.90 ± 1.26	611	245.01 ± 1.25
Female			705	259.13 ± 1.21	705	250.32 ± 1.20
Sig.				ns		ns
Crossing analysis	Value	Sig.	Value	Sig.	Value	Sig.
Purebreds difference	0.71 ± 0.07	****	25.24 ± 2.58	****	24.58 ± 2.58	****
Direct additive effect	−0.06 ± 0.03	ns	13.93 ± 1.75	****	13.99 ± 1.75	****
Heterosis contrast	0.22 ± 0.04	****	1.22 ± 0.74	*	0.95 ± 0.75	*
Heterosis %	2.60	****	2.10	*	2.07	*
Reciprocal effect	0.42 ± 0.03	****	−1.31 ± 1.18	ns	−1.70 ± 1.18	ns

* p ≤ 0.05,

**** p ≤ 0.0001, BW=Body weight, BWG=Body weight gain, W × W = White, J × J = Japanese, W × J = White male × Japanese female, J × W = Japanese male × White female, ^a,b,c,d^Different letters within a column indicate statistically significant differences, ns=Not significant, SE=Standard error

Direct additive effect

Chicks sired by WW had significantly different BWs at 6 weeks of age and BWG over the growing period (0–6 weeks) compared with chicks sired by (JJ). For both BW and BWG at these ages, the estimates of their direct additive effects were positive and statistically significant (Table-3).

Heterotic effects

At all tested ages (0, 6 weeks), significant positive heterosis contrasts (i.e., the value of the crosses was higher than the average of the parental types) were found in BW with percentages of 2.60% and 2.10%, respectively (Table-3). In addition, the heterosis percentage was positive and significant for BWG during the growth period (0–6 weeks was 2.07%).

Reciprocal effect

At an early age (hatch), the reciprocal effect was significant and positive for BW (Table-3). In contrast, no significant reciprocal effect between crosses of WW and JJ varieties was observed for BW at a late age (6 weeks) or BWG during the growth period (0–6 weeks).

## Discussion

Quantitative traits, including poultry productive performance, are influenced by environmental factors and genes. GH was one of the identified genes. In poultry species, the literature reports consistent findings concerning GH gene polymorphisms [17, 18]. According to Setiati *et al*. [5], two distinct alleles (A and B) were identified by analyzing the MspI-digested GH gene fragment of 776 bp in quail through PCR-RFLP and electrophoresis on a 3% agarose gel. Genotypes BB, AA, and AB were identified by the number of bands in their respective GH gene polymorphism: single 776 bp band (BB), two bands 539 bp and 237 bp (AA), and three bands 776 bp, 539 bp, and 237 bp (AB). The proportions of AA, AB, and BB genotypes were 41.6%, 30.5%, and 27.7%, respectively. The GH gene is known to be polymorphic based on allele frequency calculations in quail research. The GH gene’s polymorphic effects on BW were examined through selected studies on material quail.

In the genetic study, a mutation was identified in the IGF-1 gene of quails from one breed, while no difference was found between the two quail breeds (W × J and J × W) and their hybrids. El-Tarabany *et al*. [9] reported a 418 bp DNA fragment in IGF-1 amplification and deposited it in GenBank (KM278222.1). The sequences of high- and low-body-weight quails for the IGF-1 coding region were identical. El-Bayomi *et al*. [6] reported no difference in the IGF-1 gene between the chosen quail lines (high and low BW).

The linear contrasts revealed that the White purebred outperformed the Japanese purebred concerning BW at birth, BW at 6 weeks, and BWG during the phase of growth (0–6 weeks). Statistically significant differences surfaced in BW between varying quail lines/varieties throughout their growth period [19–22].

Sire-variety linear contrasts show considerable superiority of WW-sired chicks in BW and BWG. Because of these positive outcomes, it may be concluded that WW might be used as a sire variety to improve BW. Significant variations between varieties (genotypes) suggested that the additive genetic variance was high for BW and BWG. This variance was anticipated, given the evidence of significant weight gains. Analogous research has revealed that additive genes positively affect BW increase. The important effects of additive genes were reported in black and brown varieties of Japanese quails [22]. In his experiment of crossing two lines of Japanese quail chosen for high feed and water consumption from 4 to 6 weeks of age, Aboul-Seoud [21]. showed that direct additive effects were significant for BW (4.54–34.08 g) and BWG (4.70–9.00 g) at all ages tested.

In terms of BW and BWG, significant positive heterosis contrasts for BW provided evidence for the superiority of hybrids over original breeder varieties (genotypes). Furthermore, heterosis in the crossbreds showed a non-additive gene effect. Crossing a white variety with a Japanese variety would be of economic interest to improve their offspring’s growth traits (BW and BWG). Breeders can match the sire line’s growth characteristics with the higher egg production of the female line using breeding techniques. There have been reports of heterosis, or nonadditive genetic variation, in certain quail crossings in which the parents’ BWs varied significantly. Furthermore, heterosis appears to be unique to BW at certain ages rather than being a generic property of BW. When males from a randomly bred population from the same origin were crossed with females from a negligible line of Japanese quail selected for low BW at 6 weeks of age, Piao *et al*. [19] demonstrated that the heterosis effect for female BW was significant at 6 weeks of age (11.4%), but not for adult size in both males and females. During 4–6 weeks of age, two lines of Japanese quail were chosen for a crossbreeding experiment. Aboul-Seoud [21] demonstrated considerable heterosis for BW at 6 weeks of age (17.1%) and BWG at 0–6 weeks (9.1%). Crossed lines represent quails that were long-term selected for higher BW. Marks stated that significant heterosis was found for BW and that heterosis depended on age, environment, and population genetics. Furthermore, the age at which BW was examined and the hybrid combination selected affected the results of positive or negative heterosis [23].

On the other hand, crossing different lines of quail revealed negative heterosis for growth traits [24, 25]. Furthermore, Rezvannejad *et al*. [26] showed that the effect of heterosis was not significant for any BW evaluated in their study on selection for 4-week BW of lines of Japanese quail and their crosses. Non-additive genetic effects, dominance, overdominance, and epistasis are potential sources of heterosis. When combined with maternal impact, these factors have been linked to enhanced growth potential in crosses [16]. According to theory, the degree of genetic similarity between parental populations and the amount of heterosis are inversely correlated [27] and is anticipated to be correlated with the degree of cross-breed heterozygosity [28]. Thus, heterosis results from non-additive genetic effects and can be understood as both a manifestation of a certain characteristic and an overall fitness. Reproductive traits typically exhibit higher levels of heterosis than growth traits [16]. Furthermore, heterosis is the phenotypic manifestation of a multifaceted phenomenon that may entail several genetic consequences, such as dominance and epistasis. According to the fundamental quantitative genetics theory, heterosis in animal breeding should be proportional to variations in gene frequency between populations [29].

Crosses exhibit unique reciprocal effects compared to traits. Rezvannejad *et al*. [26] found that except for 4-week BW in males and 3- and 4-week BW in females, the reciprocal impact (maternal effect) in two lines of Japanese quail (high and low BW) and their crosses was significant for all BW tested at different ages (1, 2, 3, and 4 wk). In his experiment of crossing two lines of Japanese quail selected for high feed and water consumption from 4 to 6 weeks of age, Aboul-Seoud [21] reported that maternal additive effects were significant for BW (−4.28–−12.31 g) and BWG (−3.12–−5.18 g) at all ages studied (0, 2, 4, 6 weeks). It seems that in these populations, BW was influenced by both additive and non-additive genetic factors. Although Japanese quail varieties were crossed, the BW differences between them were not significant at any age [22].

## Conclusion

The GH gene exhibits two types of alleles, A and B, leading to distinct genotypic differences (AA, BB, and AB). The AA genotype showed the greatest improvement in both BW and BWG. In quails, the IGF-1 gene was identified as a mutation. A WW dam and a WW or JJ sire could be used to enhance growth traits. The hybrids exhibited superior growth traits compared to the original breeder varieties.

## Data Availability

The supplementary data can be available from the corresponding author on a request.

## Authors’ Contributions

MKS: Supervised and designed the study, drafted the manuscript, and analyzed the data. ASE: Designed the study, drafted the manuscript, and helped in the practical experiment. MHK: Designed the study, helped in the practical experiment, and drafted the manuscript. EAE: Helped in the practical experiment and drafted the manuscript. All authors have read, reviewed, and approved the final manuscript.
